# Chromatin interaction networks revealed unique connectivity patterns of broad H3K4me3 domains and super enhancers in 3D chromatin

**DOI:** 10.1038/s41598-017-14389-7

**Published:** 2017-10-31

**Authors:** Asa Thibodeau, Eladio J. Márquez, Dong-Guk Shin, Paola Vera-Licona, Duygu Ucar

**Affiliations:** 1 Department of Computer Science & Engineering, University of Connecticut, Storrs, CT USA; 2 The Jackson Laboratory for Genomic Medicine, Farmington, CT USA; 3 Center for Quantitative Medicine, University of Connecticut Health Center, Farmington, CT USA; 4 Department of Cell Biology, University of Connecticut Health Center, Farmington, CT USA; 5 Institute of Systems Genomics, University of Connecticut Health Center, Farmington, CT USA

## Abstract

Broad domain promoters and super enhancers are regulatory elements that govern cell-specific functions and harbor disease-associated sequence variants. These elements are characterized by distinct epigenomic profiles, such as expanded deposition of histone marks H3K27ac for super enhancers and H3K4me3 for broad domains, however little is known about how they interact with each other and the rest of the genome in three-dimensional chromatin space. Using network theory methods, we studied chromatin interactions between broad domains and super enhancers in three ENCODE cell lines (K562, MCF7, GM12878) obtained via ChIA-PET, Hi-C, and Hi-CHIP assays. In these networks, broad domains and super enhancers interact more frequently with each other compared to their typical counterparts. Network measures and graphlets revealed distinct connectivity patterns associated with these regulatory elements that are robust across cell types and alternative assays. Machine learning models showed that these connectivity patterns could effectively discriminate broad domains from typical promoters and super enhancers from typical enhancers. Finally, targets of broad domains in these networks were enriched in disease-causing SNPs of cognate cell types. Taken together these results suggest a robust and unique organization of the chromatin around broad domains and super enhancers: loci critical for pathologies and cell-specific functions.

## Introduction

Cell-type-specific functions of super enhancers and broad domains have been extensively studied and well established across diverse cell types and organisms^1–4^, where their distinct epigenomic profiles were instrumental in their discovery. Super enhancers are demarcated by high levels of enhancer-associated histone modification mark H3 lysine 27 acetylation (H3K27ac) and are catalogued in 86 human cell and tissue types using this mark^2^. Moreover, super enhancers have been shown to harbor Single Nucleotide Polymorphisms (SNPs) associated with the diseases of the cognate cell type, including cancer^2,4^. Pharmacological molecules have been used to effectively and specifically target super enhancer domains at oncogenes^5^, further reinforcing their significance for disease biology. Similarly, cell type-specific promoters (i.e., broad domains) are associated with expanded deposition of histone H3 lysine 4 tri-methylation (H3K4me3) mark - a signature conserved across diverse cell types (>99 in human cells) and organisms^3^. Shortening of broad domains has been observed in cancer cells at tumor suppressor genes, enabling the discovery of novel tumor suppressors^4^. Recently, super enhancers and broad domains overlapping super enhancers were shown to be more associated with chromatin interactions than their typical counterparts^6^ suggesting a unique organization of chromatin around cell-specific loci.

Chromatin structure plays a major role in governing cellular functions in a cell type- and condition-specific manner^7^. Advances in genomewide chromatin interaction profiling have shown that many regulatory elements (*i.e*., enhancers and promoters) that are distal on the linear genome map are actually in close physical proximity with each other as a result of the 3D chromatin structure^8–10^. Among these technologies, the Chromatin Interaction Analysis by Paired-End Tag Sequencing (ChIA-PET) combines chromatin immunoprecipitation with chromatin conformation capture to identify chromatin interactions that are mediated by a protein^8^, such as RNA Polymerase II (Pol2) which mediates interactions between promoters and enhancers^11^. More recently, an alternative method has been developed, HiChIP^12^, to detect protein-centric chromatin interactions^12^ using 100-fold less input material, providing an opportunity to generate such maps in primary human cells and tissues. These datasets, particularly the ones capturing protein-mediated promoter and enhancer interactions enable genomewide study of chromatin interactions between broad domains and super enhancers.

**Figure 1:**
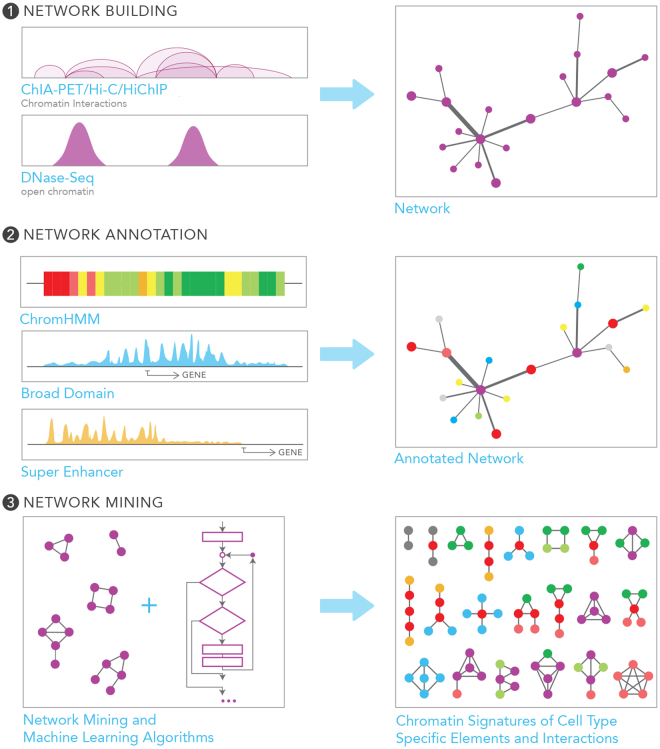
Our data analysis framework. Our three-step data analyses framework is composed of (1) network building; (2) network annotation using ChromHMM states, broad domain, and super enhancer definitions; and (3) network mining using network measures, graphlets, and machine-learning models.

This study utilizes advanced computational methods to uncover how broad domains and super enhancers interact in the 3D chromatin space, in particular, whether they are associated with distinct connectivity patterns, whether these patterns are conserved across cell types and assays, and whether they are predictive of the cell-specific nature of promoters and enhancers. For this, we built chromatin interaction networks using diverse assays (i.e., ChIA-PET, Hi-C, HiChIP) in three ENCODE cell lines: MCF-7 (breast adenocarcinoma), K562 (chronic myeloid leukemia), and GM12878 (lymphoblastoid cell line). These networks were annotated using ChromHMM states^13,14^, super enhancer^2^, and broad domain^3^ definitions in the corresponding cell types (Fig. 1). We studied interaction frequencies, network centrality measures and graphlets (i.e., small connected non-isomorphic induced subnetworks)^15^ to uncover distinct connectivity patterns associated with broad domains and super enhancers. Using machine learning models based on support vector machines (SVM)^16,17^, we showed that these chromatin connectivity patterns can effectively discriminate broad domains from regular promoters and super enhancers from regular enhancers. Our results suggest a unique and conserved chromatin organization around critical regulatory elements. Finally, we studied the clinical relevance of these annotated chromatin interaction networks by demonstrating that enhancers targeting broad domains harbor more SNPs associated to diseases of the cognate cell type.

## Results

### Chromatin interaction networks capture interactions among diverse regulatory elements

**Figure 2:**
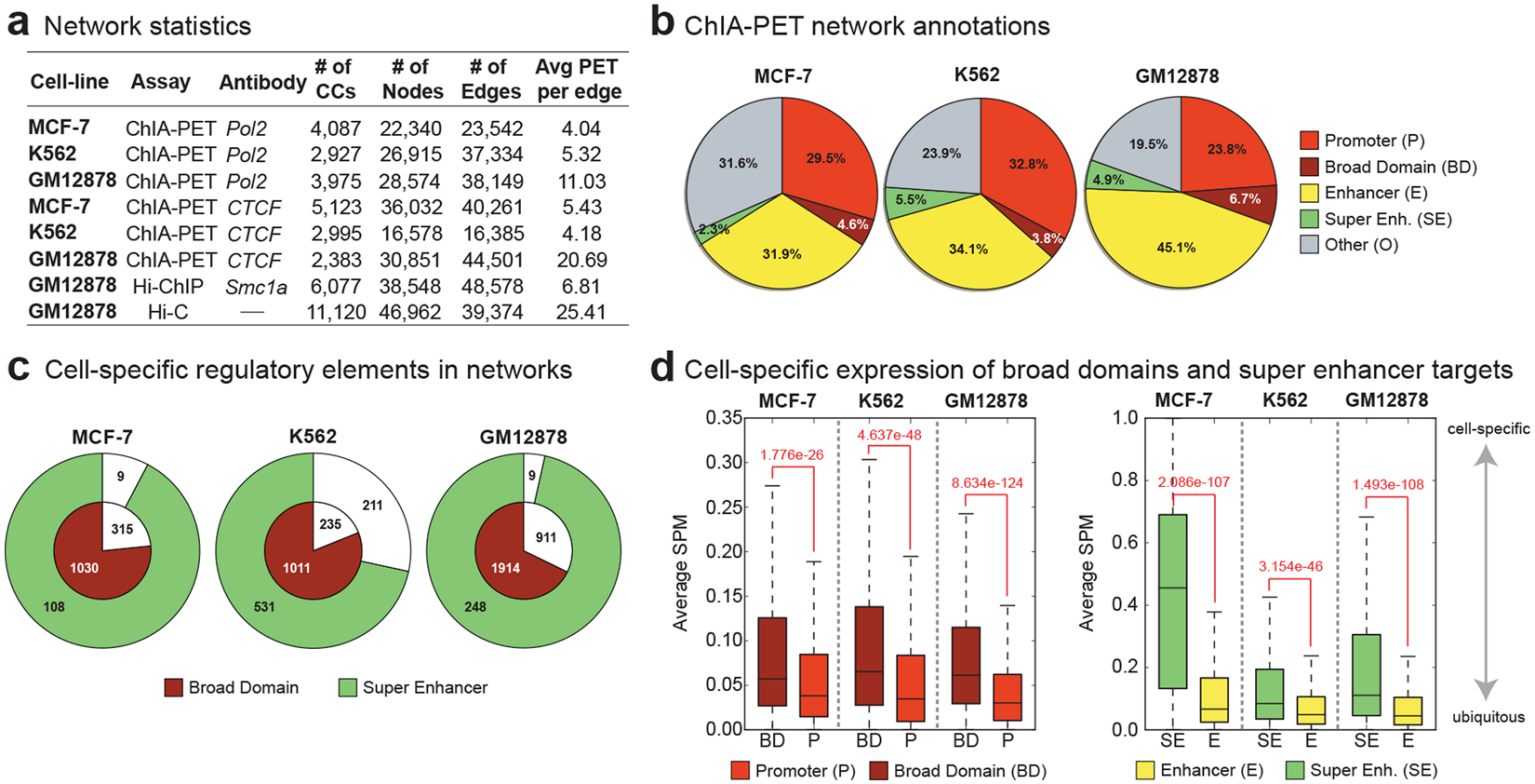
Regulatory elements in chromatin interaction networks. (**a)** Network statistics for chromatin interaction networks built from ChIA-PET, HiChIP, and Hi-C interactions in three ENCODE cell lines. **(b)** Regulatory annotations of Pol2 ChIA-PET network nodes. Note the enrichment of promoter and enhancer nodes in these networks. **(c)** Number of broad domains (inner chart) and super enhancers (outer chart) represented in ChIA-PET networks. Note that most broad domains and super enhancers in these cell lines are represented in the networks. **(d)** (Left) Cell-specific expression of genes associated to broad domains (dark red) and regular (red) promoters. Broad domain promoters are more cell-specific than regular promoters. (Right) Cell specific expression of genes associated to super enhancers (green) and regular enhancers (yellow). Super enhancer targets are more cell-specific than targets of regular enhancers.

We built chromatin interaction networks using Pol2 ChIA-PET data in three ENCODE cell lines: MCF-7 (derived from metastatic mammary grand epithelium), K562 (derived from chronic myelogenous leukemia cells), and GM12878 (lymphoblastoid cell line) and using Hi-C^18^ and HiChIP^12^ (targeting cohesion subunit *Smc1a*) data in GM12878. These networks consisted of 20–50 thousand network nodes/edges and thousands of connected components (Fig. 2a, Table S1). Next, nodes in these networks were annotated using ChromHMM states^13,14^ in conjunction with broad domain^3^ and super enhancer^2^ definitions in corresponding cell types (Methods). In Pol2 ChIA-PET networks, a majority of nodes (68–80%) overlapped promoters and enhancers, showing the utility of Pol2-mediated ChIA-PET interactions to capture interactions between regulatory elements^7^ (Fig. 2b, Supplementary Figure 1a). Majority of super enhancers and broad domains (> ~70%) were represented in these networks (Fig. 2c), in agreement with recent reports on super enhancers being more involved in chromatin interactions^6^. In comparison we also built chromatin interaction networks using CTCF-mediated ChIA-PET interactions. As expected these networks captured far fewer promoters, enhancers, broad domain and super enhancers (Supplementary Figure 2a-b, Table S1) and more insulator regions, suggesting that Pol2 ChIA-PET data is more suitable to study interactions between promoter and enhancer elements. Therefore, the rest of the ChIA-PET data analyses are conducted in Pol2 datasets. We noted that Hi-C networks include less number (~25–39% fewer) of promoters, broad domains and super enhancers compared to Pol2-associated assays (Supplementary Figure 3a-b), since Hi-C captures all DNA-DNA contacts. As previously noted^3^ genes associated with broad domains were expressed in a more cell-specific manner than genes that are active yet not associated with broad domains in the same cell type (Fig. 2d, left panel). Similarly, gene targets of super enhancers were expressed in a more cell-specific manner than the gene targets of regular enhancers in the same cell type (Fig. 2d, right panel).

### Increased interaction frequency among broad domains and super enhancers

**Figure 3:**
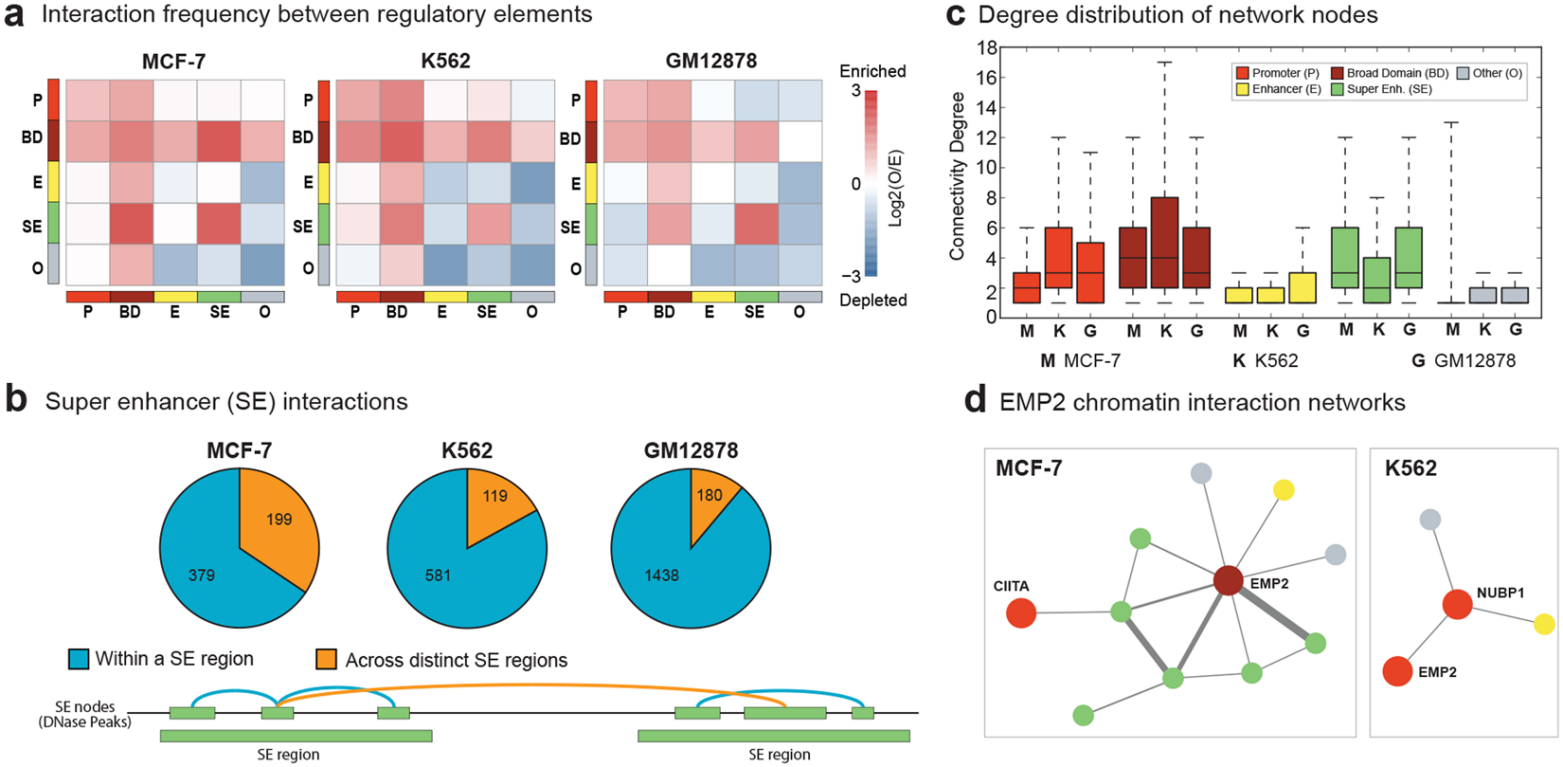
Interactions between regulatory elements in Pol2 ChIA-PET Networks. (**a**) Enrichment of interactions between pairs of annotation classes in ChIA-PET networks. Colors represent log2 ratio of observed over expected number of edges, where red represents enrichment of interactions and blue represents depletion of interactions. **(b)** (Top) Distribution of interactions within (turquoise) and across distinct (orange) super enhancer regions. (Bottom) Illustration of super enhancer nodes as defined by DNase-seq peaks within original super enhancer calls and the two different types of super enhancer interactions. **(c)** Connectivity degree distribution for different annotation classes. M, K, G represents MCF-7, K562, and GM12878, respectively. Broad domains and super enhancers are more connected on average than regular promoters and enhancers. **(d)** Example chromatin interaction networks around oncogene *EMP2*. (Left) Broad domain node associated with *EMP2* in MCF-7 is highly connected with super enhancers. (Right) Regular promoter node associated with *EMP2* is loosely connected in K562, with a single interaction with another promoter.

We calculated the frequencies of interactions between all pairs of annotations (*i.e*., broad domains, typical promoters, super enhancers, typical enhancers, and other annotations) and compared against theoretical expectations (Methods). These analyses showed that in Pol2 ChIA-PET networks, broad domains were more connected to all other nodes than theoretically expected (2.9 times more than expected) in all three cell lines (Fig. 3a, Supplementary Figure 1b). Furthermore, super enhancers interacted more frequently with broad domains (2.7–5.5 times more than expected) across the three cell types (Fig. 3a, Supplementary Figure 1b). Interestingly, super enhancer nodes also interacted more frequently among themselves (2.7–5 times more than expected), raising the possibility that distinct enhancer elements within a super enhancer region form highly interacting enhancer clusters in the 3D space. Indeed, further investigation of super enhancer-super enhancer interactions revealed that most of these (60–90%) take place within the same super enhancer region (Fig. 3b). We repeated these analyses after accounting for interactions within a single super enhancer region by representing the multiple nodes that belong to the same super enhancer domain as a single node (Methods). After this adjustment enrichment of interactions among super enhancer nodes were mostly lost (Supplementary Figure 4). Our analyses suggest that constituent enhancers within a super enhancer domain are in close proximity in the 3D chromatin space, however, these interactions do not typically span multiple distinct super enhancer domains. Finally, we noted an enrichment of interactions among promoter elements (both cell-specific and non-specific) (1.9–5.0 fold over expected, Fig. 3a). HiChIP, Hi-C, and CTCF ChIA-PET assays revealed similar interaction frequency patterns: i) high interactions between broad domains and super enhancers, ii) high interactions among constituent enhancers of super enhancer regions (Supplementary Figures 2c and 3c). Robustness of our results across assays and across cell types suggests a strong link between 3D configuration of the genome and distinct characteristics of regulatory elements. We summarized the characteristics of networks generated different assays in Table S1.

### Broad domains and super enhancers are hubs in chromatin interaction networks

Network centrality measures suggest that cell-specific regulatory elements are more connected and exhibit hub-like connectivity in these networks in comparison to their typical counterparts (Fig. 3c, Supplementary Figure 5). On average, promoters were connected to 2.63, 4.21, and 3.56 other nodes in MCF-7, K562, and GM12878 Pol2 ChIA-PET networks respectively, whereas the corresponding values for broad domains were 5.03, 5.83, and 4.49 (one-sided Wilcoxon test p-values < 4.4.e-32 for all three cell lines). The increased connectivity of broad domain promoters taken together with their frequent interactions with super enhancers might be essential in maintaining their robust and increased gene expression patterns. On the other hand, enhancer nodes were connected to an average of 1.81, 1.90, 2.25 other nodes respectively in MCF-7, K562 and GM12878, whereas super enhancer nodes averaged 4.61, 3.27, and 4.22 connections respectively (one-sided Wilcoxon test p-values < 5.1e-114 for all comparisons). Our results also revealed a higher betweenness score for broad domains relative to non-specific promoters (Supplementary Figure 5) suggesting that broad domains act as connectors in the networks. For example, Fig. 3d shows Pol2 ChIA-PET network involving the *EMP2* oncogene that is upregulated in invasive breast cancer patients^19^. In the breast cancer cell line MCF-7, *EMP2* maps to a broad domain node and is connected to multiple super enhancers (Fig. 3d, left panel), which are also connected to each other. In contrast, in K562 where *EMP2* is an active yet non-specific promoter, this locus is connected differently and less densely (Fig. 3d, right panel). On the other hand, this locus was repressed in GM12878, and was not represented in the corresponding networks. This example illustrates that connectivity of a locus in the 3D chromatin reflects the functional importance of that region in the cognate cell type. Our analyses revealed that cell-specific regulatory elements are connected more frequently in the 3D genome in comparison to their non-specific counterparts.

### Broad domains and super enhancers have unique connectivity patterns

**Figure 4:**
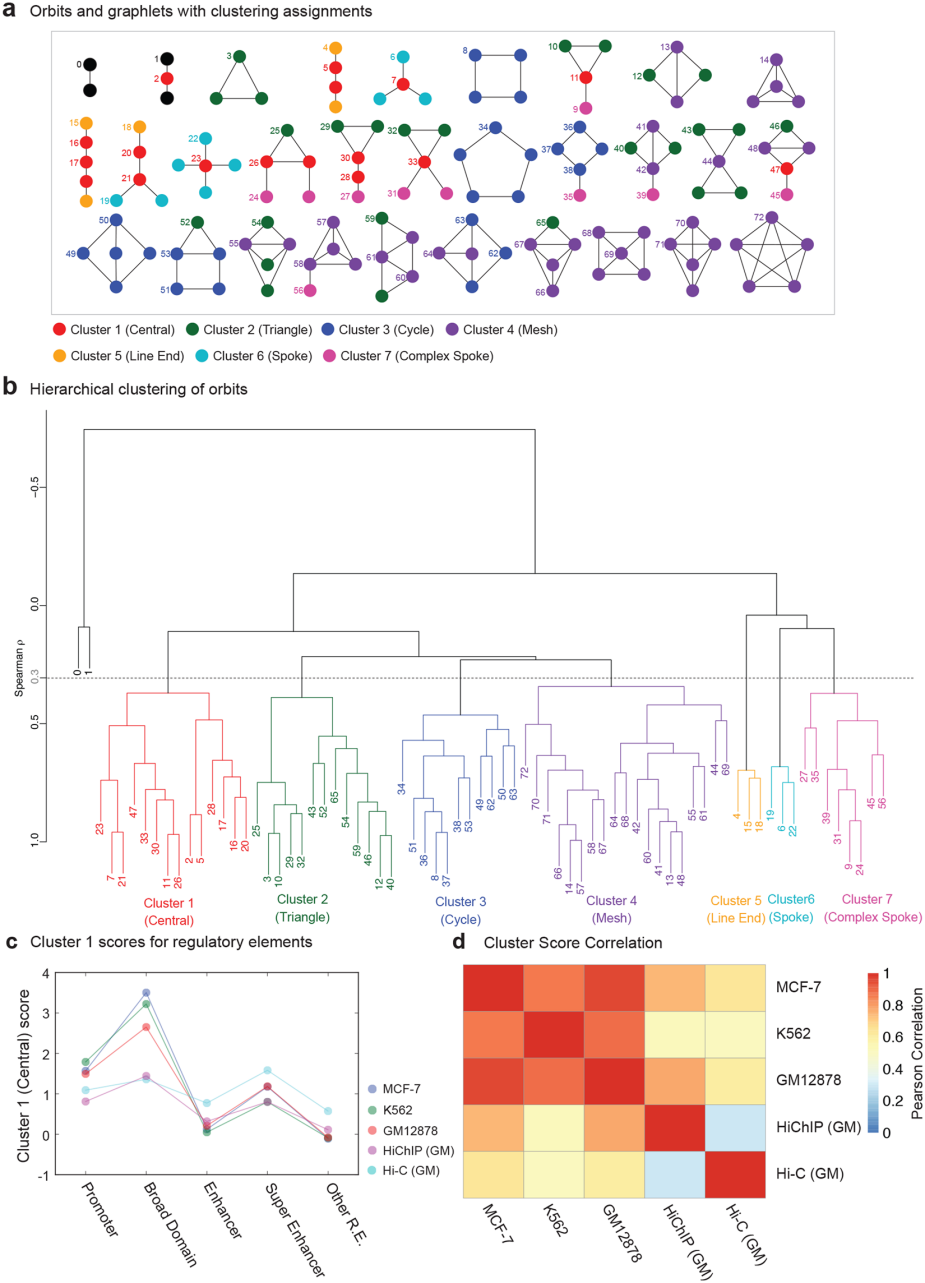
Cell-specific regulatory elements are associated with distinct network patterns. (**a)** Representation of 73 orbits used to drive orbit cluster signatures. Orbit nodes are color-coded with respect to their cluster assignments from **(b)**. For example red nodes represent central nodes that are in cluster 1. **(b)** Hierarchical clustering of 73 orbits after pooling data from all Pol2 ChIA-PET networks. Seven distinct clusters are identified based on the topology of network graphlets. **(c)** Trimmed mean Cluster-1 score (central nodes) for different annotations in ChIA-PET, Hi-ChIP, and Hi-C networks. Broad domain nodes have the highest C1 score in these networks. **(d)** Pairwise correlations (Pearson’s coefficient) of 7 cluster scores computed over each annotation between all network pairs. Note the high correlation across different assays and cell types (except for Hi-C), suggesting that network connectivity patterns are non-stochastic.

Chromatin interaction networks offer the opportunity to explore higher-level chromatin connectivity patterns (*i.e*., network motifs) beyond immediate interactions. However, enumerating all possible configurations in a large network is computationally intractable (subgraph isomorphism problem). To effectively and systematically uncover chromatin interaction patterns associated with cell-specific regulatory elements, we utilized graphlets^15^. Graphlets are small, connected, and non-isomorphic (*i.e*., topologically different) subnetworks within a large network that enables systematically studying and quantifying the local network structure around a node of interest. Topologically distinct nodes within a graphlet are referred to as orbits. We studied the local structure of chromatin interaction networks using all possible graphlets composed of two to five nodes, which encompasses 73 orbits (Fig. 4a). For each node an orbit signature vector is compiled by counting the number of times each node possessed the local structure of the 73 orbits. Orbits were then clustered to account for their topological similarities (Methods) revealing seven major orbit clusters that represent topologically distinct types of orbits (hierarchical clustering, Spearman coefficient cutoff = 0.3) (Fig. 4b). For example, Cluster 1, C1, is composed of orbits occupying a central position across various graphlets (red nodes in Fig. 4a). Therefore, a node that has a high C1 score occupies a central position in its chromatin interaction network. For each network node we calculated their orbit cluster scores (n = 7 scores), which allowed us to systematically assess recurrent chromatin interaction patterns associated with specific regulatory elements. Promoters (regular and broad domain promoters) in general had higher C1 scores than enhancers, indicating that they are more likely to be at the center of chromatin interaction networks (Fig. 4c). Broad domains held the most central positions in these networks, in agreement with their high connectivity degree (Fig. 3c). On the other hand, among the enhancer elements, super enhancers were more centrally located (*i.e*., higher C1 score) than regular enhancers (Fig. 4c). Furthermore, super enhancer nodes exhibited more clique-like structures (e.g. triangle, cycle, and mesh patterns) than typical enhancers as evident from their higher C2, C3, and C4 scores (Supplementary Figure 6). Strikingly, orbit cluster scores of different functional elements showed very consistent patterns across cell types and across different assays (Fig. 4d), suggesting that these connectivity patterns are not stochastic and have functional relevance. These results suggest that cell type-specific regulatory elements have unique connectivity patterns and they tend to be central and tend to form tightly connected subnetworks in the 3D chromatin space.

### Network connectivity patterns are predictive of cell-specific activity

**Figure 5:**
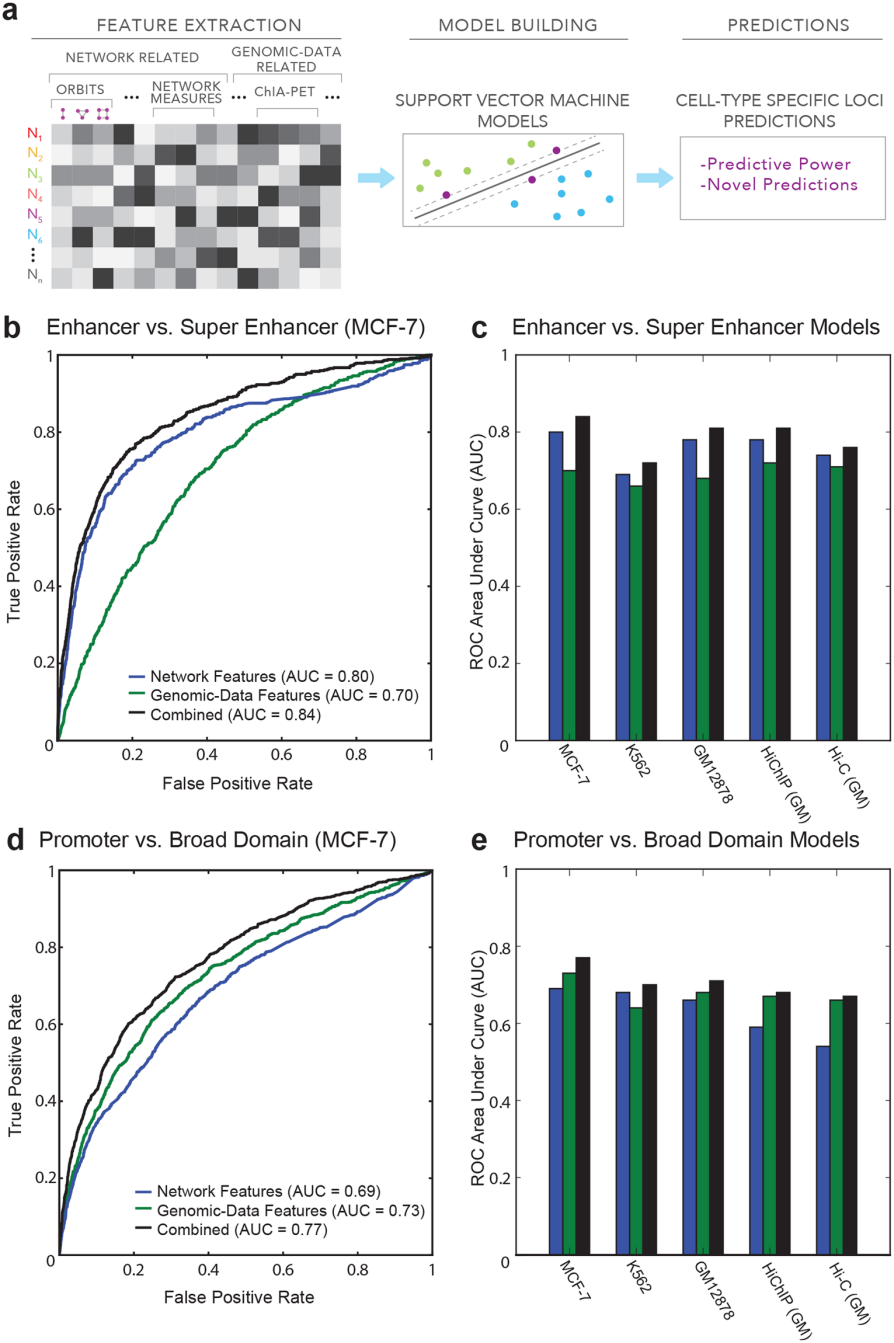
Network connectivity patterns are predictive of cell-specific activity. (**a)** Summary of our machine-learning framework. **(b)** Receiver Operating Characteristic (ROC) curves for SVM models discriminating enhancers from super enhancers in MCF-7. **(c)** Receiver Operating Characteristic (ROC) area under the curve (AUC) values for enhancer vs. super enhancer predictions over all networks. **(d)** ROC curves for SVM models separating promoters from broad domains in MCF-7. **(e)** Receiver Operating Characteristic (ROC) area under the curve (AUC) values for promoter vs. broad domain prediction over all networks.

To determine whether chromatin connectivity patterns of regulatory elements can be predictive of their cell-specific activity, we used machine-learning models based on support vector machines (SVM) (Fig. 5a). Each network node was represented using two types of data features: (1) network related and (2) genomic-data related and we built SVM-based^16,17^ classification models to discriminate i) broad domains from regular promoters and ii) super enhancers from regular enhancers. The discriminative power of these models was quantified using receiver operating characteristic (ROC) curves and area under these curves (AUC), where a perfect predictor (*i.e*., one that only identifies a ‘real’ broad domain as a broad domain) has an AUC score of 1.

SVM models efficiently discriminated super enhancers from regular enhancers with high accuracy (Accuracy = 0.91, 0.74, 0.84 at 0.2 probability threshold, AUC = 0.84, 0.72, 0.81 in MCF-7, K562, and GM12878 Pol2 ChIA-PET networks respectively) (Fig. 5b-c, Supplementary Figure 7). Similarly, these models were also effective in discriminating broad domain promoters from regular promoters (AUC scores = 0.77, 0.70, 0.71 for MCF-7, K562, and GM12878 respectively) (Fig. 5d-e, Supplementary Figure 8). Furthermore, both analyses revealed that integration of network related features (*e.g*., orbit cluster scores) with other genomic features improved the predictive ability of these models, suggesting that chromatin interaction networks and connectivity patterns harbor functional and non-redundant information. Prediction of super enhancers in HiChIP and Hi-C was as effective (AUC = 0.81 and 0.76 respectively) (Supplementary Figure 9). Similar results for broad domain classification models were achieved from Hi-C and HiChIP data (Supplementary Figure 10), although the relatively smaller impact of network features in these analyses suggests that Pol2-mediated interactions may be better suited for capturing network patterns associated with promoters. Precision-recall curves (Supplementary Figures 7–10) further emphasized the value of integrating network and genomic data features and the ability of these datasets to predict cell-specific regulatory elements.

The most predictive data features in these models were obtained using forward selection (Supplementary Figures 11 and 12), which uncovered consistent ranking of discriminatory features. For broad domain predictions, the two most predictive features were node size and network features related to their centrality (Supplementary Figure 11). Further investigation revealed that indeed broad domains are associated with expanded chromatin accessibility around their promoters (Supplementary Figure 13), in agreement with the expanded H3K4me3 deposition observed at these loci^3^. On the other hand, the most predictive features for super enhancers were related to their clique-like connectivity (*i.e*., mesh) and high degree, reinforcing the importance of tight connectivity around super enhancers (Supplementary Figure 12). In summary, results from these analyses showed that network connectivity patterns of a regulatory element is predictive of its importance for regulating critical cellular functions in that cell type. Prediction results from our models can be found in Table S2.

### Enhancers targeting broad domains in interaction networks are enriched in disease-causing SNPs

**Figure 6:**
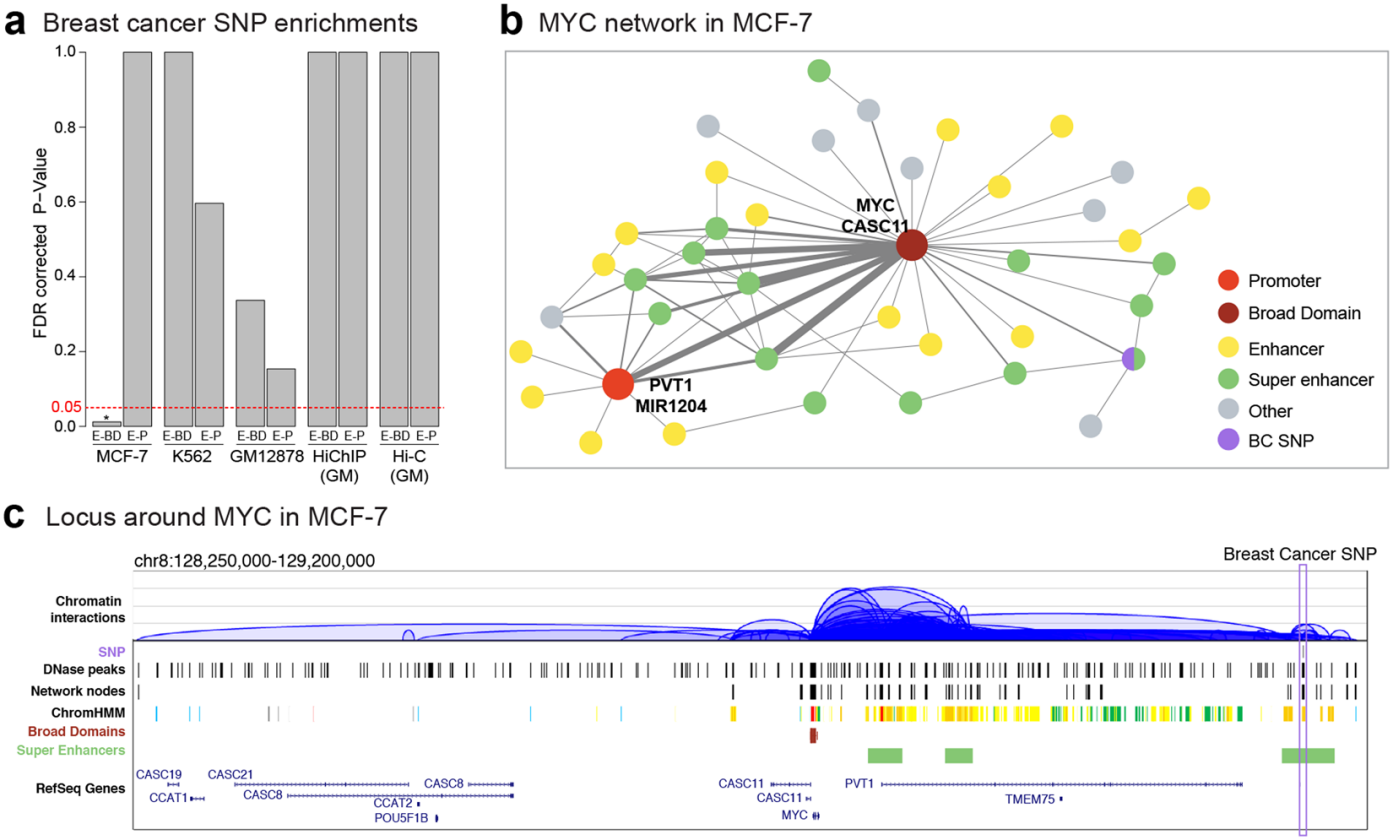
Enhancers targeting broad domains are enriched in variants associated to the diseases of the cognate cell type. (**a**) Enrichment p-values for breast-cancer SNPs at enhancers targeting promoters (E-P) and enhancers targeting broad domain promoters (E-BD) in different cell types. Breast-cancer associated SNPs are significantly enriched in enhancers targeting BDs in MCF-7 breast cancer cell line. **(b)** Example MCF-7 network representing breast-cancer associated SNP rs1121946 in a super enhancer node targeting the *MYC* broad domain promoter. **(c)** Genome browser representation of the locus around *MYC*. Note that network representation is effective in detecting connectivity patterns.

Previous studies have established that disruption of enhancer activity is associated with pathologies, particularly at cell-specific enhancers^4,14^. Here we investigate whether chromatin interaction networks can be utilized in further prioritizing loci associated to disease-causing genetic variants and in identifying their gene targets. To test this, we calculated the enrichment of NHGRI GWAS SNPs^20^ in enhancers targeting regular promoters and enhancers targeting broad domains. Our analyses revealed that in MCF-7, enhancers (regular and super) targeting broad domains were significantly enriched in breast-cancer associated SNPs (Fig. 6a, Benjamini-Hochberg adjusted p-value = 0.012). This includes 7 out of 1757 enhancer/super enhancer nodes harboring breast cancer SNPs (Table S3), one of which is a super enhancer and targets the promoter of the *MYC* oncogene (Fig. 6b-c) that is constitutively expressed in many cancers, including breast and blood cancers^21^. In breast cancers, *MYC* de-regulation is associated with breast cancer development and poor prognosis^22^. The locus around *MYC* is active and represented in the chromatin interaction networks in all three cell types, however connectivity around this locus was different in these cells. In the MCF-7 network, *MYC* is a broad domain node and connected to multiple enhancer and super enhancer nodes, one of which harbors rs1121946 SNP that is in high linkage disequilibrium (LD) with index SNP rs11780156 (r^2^ > 0.7), a known breast-cancer associated variant (Fig. 6b-c). Our network representation revealed that although this SNP is within an enhancer domain that is 414 kb away from the *MYC* promoter, it is directly connected to *MYC*’s promoter in MCF-7 networks, emphasizing the importance of studying disease-associated SNPs using 3D chromatin interaction networks. Similar to the enrichment of breast cancer associated SNPs in MCF-7, we observed that in GM12878, enhancers targeting broad domains were enriched in SNPs associated with immune-related disorders, such as Systemic Lupus and Crohn’s disease in comparison to enhancers targeting regular promoters (Table S4). This analysis revealed the utility of chromatin interaction networks to uncover and prioritize non-coding loci associated with pathologies as well as their gene targets.

## Discussion

In this study, we showed that broad domains and super enhancers have distinct connectivity patterns in chromatin interaction networks that are conserved across cell types and can be captured using diverse assays. Broad domains tend to be central nodes in these networks and are frequently targeted by multiple constituent enhancers that belong to a super enhancer domain. A recent study employed CRISPR/Cas9 to delete enhancers and super enhancers in mouse embryonic stem cells (mESCs) and suggested that super enhancer clusters act in a redundant manner to fine tune the expression of their target genes^23^. Our observations from chromatin interaction networks provide an explanation for this redundancy. We observed high connectivity among super enhancer elements and high connectivity between broad domains and constituent super enhancers. This tightened connectivity among cell-specific regulatory elements ensures that the activity of super enhancer target genes (likely to be a broad domain) is robust to the disruption of any single enhancer element within the enhancer cluster. Such a tightened connectivity pattern might also be critical in establishing and maintaining robust (i.e., low variation) expression patterns associated with broad domain genes^3^. Our machine learning models showed that connectivity patterns of super enhancers and broad domains are predictive of their cell-specific nature.

Recent studies revealed that regulatory elements with frequent chromatin interactions (i.e., hubs) are enriched in super enhancers and harbor more GWAS SNPs^6,24,25^. Our study is in alignment with these findings where we observed increased connectivity for super enhancer nodes in comparison to regular enhancers using three different assays (ChIA-PET, Hi-C, and HiChIP) and in three different cell types (K562, MCF-7, GM12878). Our study furthered our knowledge on how super enhancers are connected in these networks and revealed that super enhancers form clique-like structures and typically connect to broad domains and other super enhancer elements within the same domain. Broad domains were associated with more chromatin interactions compared to typical H3K4me3 domains^6^. Our study established that in comparison to regular promoters, broad domains had more interactions overall. Moreover, these interactions were especially connecting them to super enhancers. Increased targeting of broad domains by super enhancers likely ensures robust and increased expression of these important genes in cognate cells. Furthermore, we showed that enhancers targeting broad domains in chromatin interaction networks harbor disease-associated SNPs, further reinforcing the importance of studying these genes and their regulators in the context of human diseases.

A challenge we faced in our analyses was the difficulty of representing super enhancers that span long genomic regions (10–20 kb) in our networks, which would harbor more interactions merely due to their genomic coverage. We overcame this challenge by defining the network nodes using open chromatin regions (i.e., DHS sites), which represent putative active regulatory elements. This methodology also enabled the study of individual enhancer elements within a super enhancer domain. We observed that a super enhancer element typically is not a single and expanded regulatory element but a combination of constituent active enhancers that are in close proximity to each other in both linear and 3D space, and they frequently interact with one another and with their target gene to regulate that gene’s expression levels (see examples of such networks in Figs 3d, and 6b). Our analyses also showed that although members of a super enhancer domain heavily interact among themselves, these enhancers typically do not interact with other super enhancer domains.

In conclusion, our study uncovered the unique chromatin interaction patterns around loci that are critical for cellular functions and disease etiology by taking advantage of network theory and machine learning models. By building interaction networks we were able to study complex network patterns (such as graphlets) and to systematically quantify differences in the connectivity of different regulatory elements. Our findings revealed that chromatin connectivity patterns around super enhancers and broad domains are non-stochastic and conserved across cell types and can be captured via different assays. However, we acknowledge the caveat that genome-wide chromatin interaction maps analyzed here are generated from millions of cells. Therefore it is not possible to dissect whether the connectivity patterns observed for super enhancers and broad domains take place in individual cells. Advances in single-cell chromatin interaction profiling techniques will be essential in studying these patterns at the single cell level. As a step towards this direction, Hi-ChIP^12^ a recent technique significantly reduced the input material required to profile protein-mediated chromatin interactions: a 100 fold decrease from 100 million cells to 1 million cells. Similarly, recent developments in single-cell Hi-C profiling techniques open the doors to studying cell-to-cell-heterogeneity for DNA-DNA interactions^26^. With these technological advances, we expect more chromatin interaction data to be generated from human cells in healthy and disease states at the single-cell resolution in the near future. Advanced computational methods we present in our study will be critical in furthering our understanding on how chromatin interactions might relate to establishing and maintaining critical cellular functions and how changes in these interactions might be associated with pathologies.

## Methods

### ChIA-PET data analyses and network construction

Pol2 and CTCF ChIA-PET chromatin interactions called using ChIA-PET Tool^27^ were obtained from^28,29^. Accession numbers for these datasets are as follows: MCF-7 (GSE39495), K562 (GSE39495), and GM12878 (GSE72816). ChIA-PET Tool^27^ interactions were preferred over the alternative Mango^30^, since calls obtained via Mango were sparse and did not include as many broad domain or super enhancer interactions (Table S1). To minimize the number of false positive interactions, we instead filtered interaction calls using QuIN^31^, by selecting only those having both anchors overlapping DNase I hypersensitive sites (DHS) defined from DNAse-seq open chromatin peaks, which reduces false positive calls and likely captures active regulatory loci. These peaks where then used to define the nodes of the interaction networks, which were constructed using QuIN^31^. MCF-7, K562, and GM12878 open chromatin peaks were called using MACS2 software^32^ (version 2.1) after pooling replicates (GSE32970 and GSE29692^28^). For network building, we used 250 bp extension and removed inter-chromosome interactions and edges spanning distances greater than 1 Mb. Network nodes were first annotated using ChromHMM states^13,14^. If a node overlapped with multiple ChromHMM annotations, we applied the following priority schema to dissolve ambiguities in annotations due to the genomic segmentation framework in ChromHMM: 1) enhancers/promoters 2) insulators 3) poised promoters 4) repressed elements 5) transcribed elements 6) low signal. If a node was annotated both as a promoter and an enhancer, known transcription start site (TSS) definitions were used to define promoter nodes found within 2 kb of a known TSS. Broad domains and super enhancers annotations were then assigned respectively to promoter and enhancer nodes using the previously defined broad domain^3^ and super enhancer^2^ regions in corresponding cells. If a broad domain was found to overlap multiple promoter nodes, the promoter node with the largest overlap with the broad domain in terms of base pairs (bps) was assigned as the broad domain node. In the case of ties, both promoters were labeled as broad domain nodes.

### Processing and analyses of Hi-C and HiChIP data

We called interaction pairs from HiChIP^12^ (GSE80820) (targeting cohesion subunit *Smc1a*) and Hi-C^18^ (GSE63525) data in GM12878 cell line. Valid interaction pairs from HiChIP biological and technical replicates were pooled and filtered by extending 250 bp in both directions, keeping only the pairs with both ends overlapping a DNase-seq peak. Significant HiChIP interactions between peaks were called based on the hyper-geometric distribution as described in^27^ and filtered using the Benjamini-Hochberg procedure (FDR < 0.05). Significant Hi-C intra-chromosomal interactions at 1 kb resolution were identified using in-house software implemented based on the HiCCUPS method^18^. For each contact, expected values based on donut, vertical, horizontal and lower left filters were calculated using parameters P = 20 and W = 40 to calculate a P-value based on the Poisson distribution and filtered based on the Benjamini-Hochberg procedure (FDR < 0.025). A more stringent FDR cutoff is used for Hi-C data to make it more comparable with other assays, since Hi-C data were more deeply sequenced than the others. The intersection of these four filters was used to identify the final set of contacts. Network construction was performed similar to ChIA-PET data using 0 bp and 1,250 bp extension parameters for HiChIP and Hi-C networks, respectively. These values where chosen to account for differences in read extension introduced in interaction calling steps. HiChIP edges were further filtered to remove edges with less than 4 supporting valid pairs.

### Processing and analyses of RNA-seq data for cell-type specificity

RNA-seq data was obtained from the ENCODE project repository for all available cell lines and adult tissues^28,33^. These data were filtered to exclude samples that were generated under an experimental treatment or audited for quality concerns. This resulted in 23 cell and tissues types (including GM12878, K562, and MCF-7) that were used to quantify the degree of cell-specificity of gene expression levels. For this, we calculated the cell-type specificity measure (SPM)^34^ for each gene in the three cell types. SPM was used to quantify cell-specificity of genes associated to regular and broad domain promoters, as well as target genes of regular and super enhancers. For super enhancers and enhancers, we considered both nearest TSS definitions (up to 10 kb) as well as promoter targets identified from ChIA-PET networks. Network targets were chosen by selecting promoter/broad domain targets of up to 4 edges using breadth first search between the enhancer and promoter node, stopping at the first promoter node found. If a network node was associated with multiple genes, the average SPM measure of all associated genes was used.

### Interaction frequency between functional annotations

We calculated the enrichment of interactions between every pair of annotations (e.g., promoter versus enhancer nodes) by calculating a theoretical expectation of interaction frequency and by comparing against the observed interaction frequency for this annotation pair. More formally, expected interaction frequency between all nodes annotated as “*A*” and all nodes annotated as “*B”* is calculated as:

1 $${\rm{Expected}}(A,B)=\sum _{{C}_{i}}|{E}_{{C}_{i}}|\frac{\sum _{{a}_{j}\in {C}_{}}\sum _{{b}_{j}\in {C}_{i}}r(\text{gd}({a}_{j},{b}_{j}))}{\sum _{{n}_{j}\in {C}_{i}}\sum _{{n}_{k}\in {C}_{i},j < k}r(\text{gd}({n}_{j},{n}_{k}))}\quad r(x)=\{\begin{array}{cc}1 & {\rm{if}}\,x=1000000\\ 0 & {\rm{otherwise}}\end{array}$$

where *C*_*i*_ represents the set of all nodes in chromosome *i*, *|E*_*Ci*_ *|* represent the number of observed edges within the node subset *C*_*i*_ and *gd(x*, *y)* is the genomic distance from node *x* to node *y*. The expected frequency obtained corrects for both analyzing only intra-chromosomal interactions as well as the limitation that connected nodes within the network are within 1 Mb. For nodes with the same annotation, the expected value was adjusted accordingly to account for two nodes being selected from the same set.

To study whether super enhancer interactions are restricted within a domain, we generated interactions networks again by merging super enhancer nodes that belong to the same super enhancer domain and representing the whole domain as a single node in the network. Then we counted the number of edges that are connecting these merged nodes with the rest of the nodes in the network and repeated the interaction enrichment analyses.

### Centrality measures

Network metrics were calculated using QuIN^31^. Connectivity degree refers to the number of edges connected to a node. Closeness centrality was computed as:

2 $${\rm{Closeness}}(v)=\frac{1}{\sum _{u\in {N}_{c}}{\rm{nd}}(u,v)}$$

where *nd* is the network distance (*i*.*e*., number of edges) between nodes *u* and *v*, and *N*_*c*_ represents the set of nodes within the connected component containing *v*. A connected component is defined as a collection of nodes where every node pair is connected to each other through some path (*i*.*e*., a sequence of adjacent edges) such that nodes are not connected to other nodes outside this collection. Harmonic centrality was computed as follows:

3 $${\rm{Harmonic}}(v)=\sum _{u\in {N}_{c},u\ne v}\frac{1}{{\rm{nd}}(u,v)}$$

Finally, betweenness centrality was calculated as:

4 $${\rm{Betweenness}}(v)=\sum _{u\ne v\ne x\in {N}_{c}}\frac{| {\rm{sp}}(u,x,v)| }{| {\rm{sp}}(u,x,x)| }$$

where *sp(u*, *x*, *v)* denotes all shortest paths between nodes *u* and *x* that include node *v*. Normalized centrality measures of a node were calculated with respect to the size of the network component |*N*_*c*_|.

Comparisons of connectivity degree measures of promoters to broad domains and super enhancer to enhancers were performed using the Wilcoxon rank sum test. The Wilcoxon rank sum test is a non-parametric test that does not assume normally distributed measurements. Corresponding n-values for each annotation subset and network are reported in Table S1.

### Graphlet and orbit scores

Graphlets are small, connected, and non-isomorphic (*i.e*., topologically distinct) subnetworks components that can be used to decompose and describe a large network^15^. Topologically distinct nodes of a graphlet are referred to as orbits. There exist 73 possible orbits for all graphlets of size 2–5, which were counted using the Cytoscape plugin GraphletCounter^35^. For each ChIA-PET network node, a vector of 73 orbit counts was obtained. The proportion of each orbit with respect to all 73 orbits counted was calculated to normalize each vector. To identify orbits that are topologically similar, we performed hierarchical clustering based on Spearman rank correlation coefficient on orbit scores of ChIA-PET network nodes. Seven orbit clusters were obtained at a Spearman cutoff of 0.3 (Fig. 4b) and visually interpreted and labeled based on their connectivity patterns after excluding orbits 0 and 1, which were present and have similar scores for all nodes. Finally, scores for each orbit clusters is calculated as follows:

5 $$C{S}_{ki}={{\rm{\max }}}_{j\in {C}_{k}}(\frac{{O}_{ij}-{\mu }_{j}}{{\sigma }_{j}})$$

where *CS*_*ki*_ denotes the score of node *i* for cluster *k*, *O*_*ij*_ denotes the orbit score for node *i* and orbit *j*, *C*_*k*_ denotes the set of orbits in cluster *k*, and *μ*_*j*_ and *σ*_*j*_ represent the mean and standard deviation of orbit *j*’s score over all nodes.

### Support Vector Machine models

We used support vector machines (SVMs) due to their efficacy in handling high dimensional and noisy data^36^, which is the case in genomics data. SVMs are supervised learning algorithms that effectively handle classification problems^37^ by mapping each example as a point in a higher dimensional data space and identifying a hyperplane that separates classes by the widest margin. To build SVM models, we extracted two types of data features we extracted for each network node: (1) network related and (2) genomic-data related. We extracted sixteen network related features, which included network measures (both raw and normalized by component size) (n = 8), connected component size, and graphlet cluster scores (n = 7). For genomic-data related features, we extracted and used seven features: open chromatin peak length in bps (equivalent to node size in our networks), the ratio of a node’s size to the average size of all nodes connected to it, the average PET of a node’s edges, and gene definition-related features (n = 4), which include the distance to the closest upstream and downstream TSS and the direction of transcription for these two genes. SVM^16,17^ models were trained with a radial basis function (RBF) kernel using scikit-learn Python libraries^38^. Grid search was used to tune hyper-parameters of the SVM models (*C* and *gamma*). For feature ranking, we implemented forward feature selection using a greedy algorithm that implemented a stepwise addition of features to the model, keeping the best-performing feature in the model for each round until all features were included. Matthews correlation coefficient (MCC) was used as a performance measure both during hyper-parameter tuning and feature selection analyses, since this measure is effective for unbalanced class sizes (i.e., 500–1400 super enhancer nodes compared to 7000–13000 enhancer nodes).

### GWAS SNP enrichments

We tested for enrichment of GWAS SNPs in enhancer loci targeting regular and broad domain promoters using GREGOR^39^. Index SNPs from the GWAS catalogue^20^ were used for enrichment analyses (n = 657 phenotypes) along with the SNPs that are in high linkage disequilibrium (LD) with the index SNPs (R^2^ > = 0.7). Benjamini-Hochberg FDR method was used for multiple hypothesis correction of enrichment p-values.

### Availability of data and methods

Datasets and scripts are available on the Ucar Lab github website: https://github.com/UcarLab/CellSpecificNetworks.

## Electronic supplementary material

Supplementary Figures

Table S1

Table S2

Table S3

Table S4
