# Supplementary material for: Chromatin interaction networks revealed unique connectivity patterns of broad H3K4me3 domains and super enhancers in 3D chromatin
Source: Sci Rep. 2017 Oct 31;7:14466. doi: 10.1038/s41598-017-14389-7 (PMC5663946; doi:10.1038/s41598-017-14389-7)
Supplement: Supplementary file 1 — Supplementary Figures [file 41598_2017_14389_MOESM1_ESM.pdf]

Chromatin interaction networks revealed unique connectivity patterns of broad H3K4me3 domains and super enhancers in 3D chromatin

Asa Thibodeau[1], Eladio J. Márquez[2], Dong-Guk Shin[1], Paola Vera-Licona[3,4,5]* and Duygu Ucar[2,5]*

[1]Department of Computer Science & Engineering, University of Connecticut, Storrs, CT, USA.

[2]The Jackson Laboratory for Genomic Medicine, Farmington, CT, USA.

[3]Center for Quantitative Medicine, [4]Department of Cell Biology, [5]Institute of Systems Genomics, University of Connecticut Health Center, Farmington, CT, USA.

*Corresponding authors: veralicona@uchc.edu, duygu.ucar@jax.org

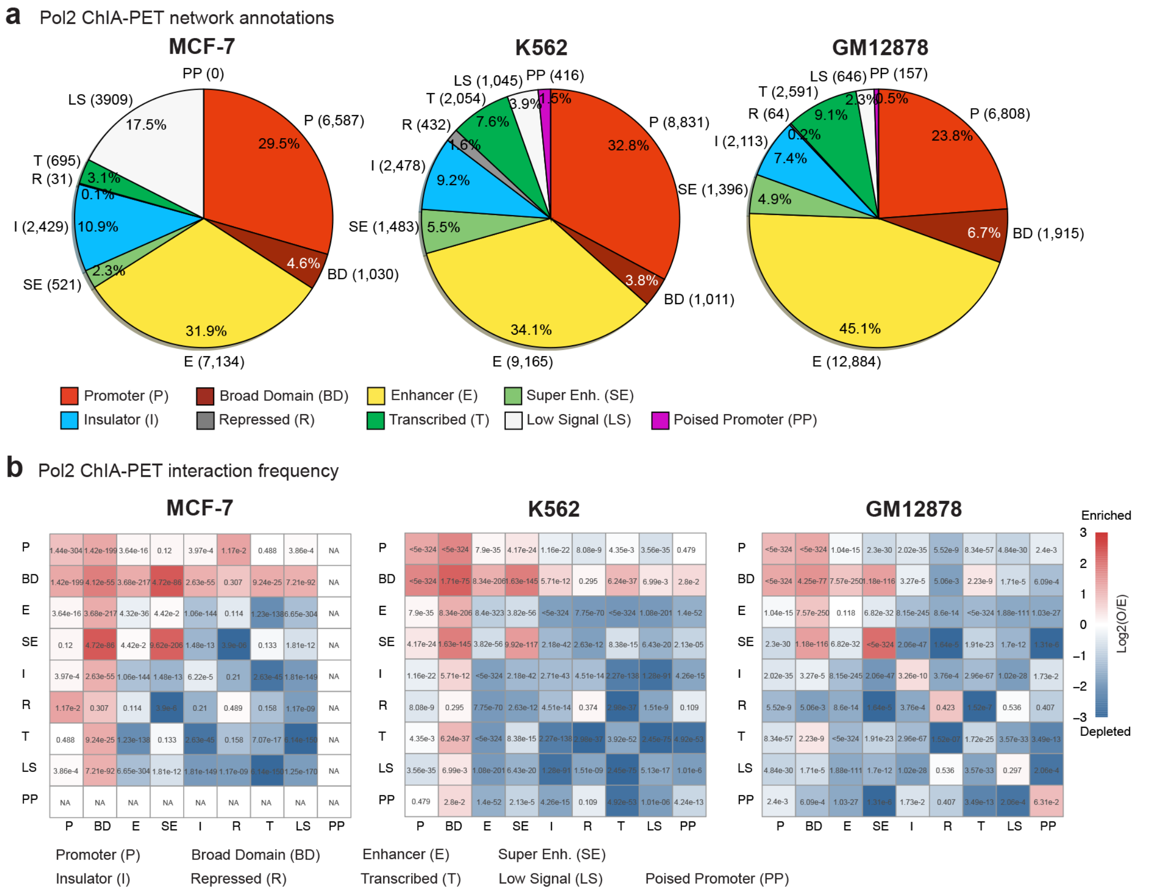

Supplementary Figure 1. Representation of regulatory elements and interaction frequencies in Pol2-mediated ChIA-PET networks. (a) Distribution of different annotations. **(b)** Interaction frequency matrices between pairs of annotation classes based on the log2 ratio of observed over expected number of edges between nodes with the respective annotations. Values within each cell represent one tailed binomial test p-values (greater than or less than hypothesis depending on the ratio).

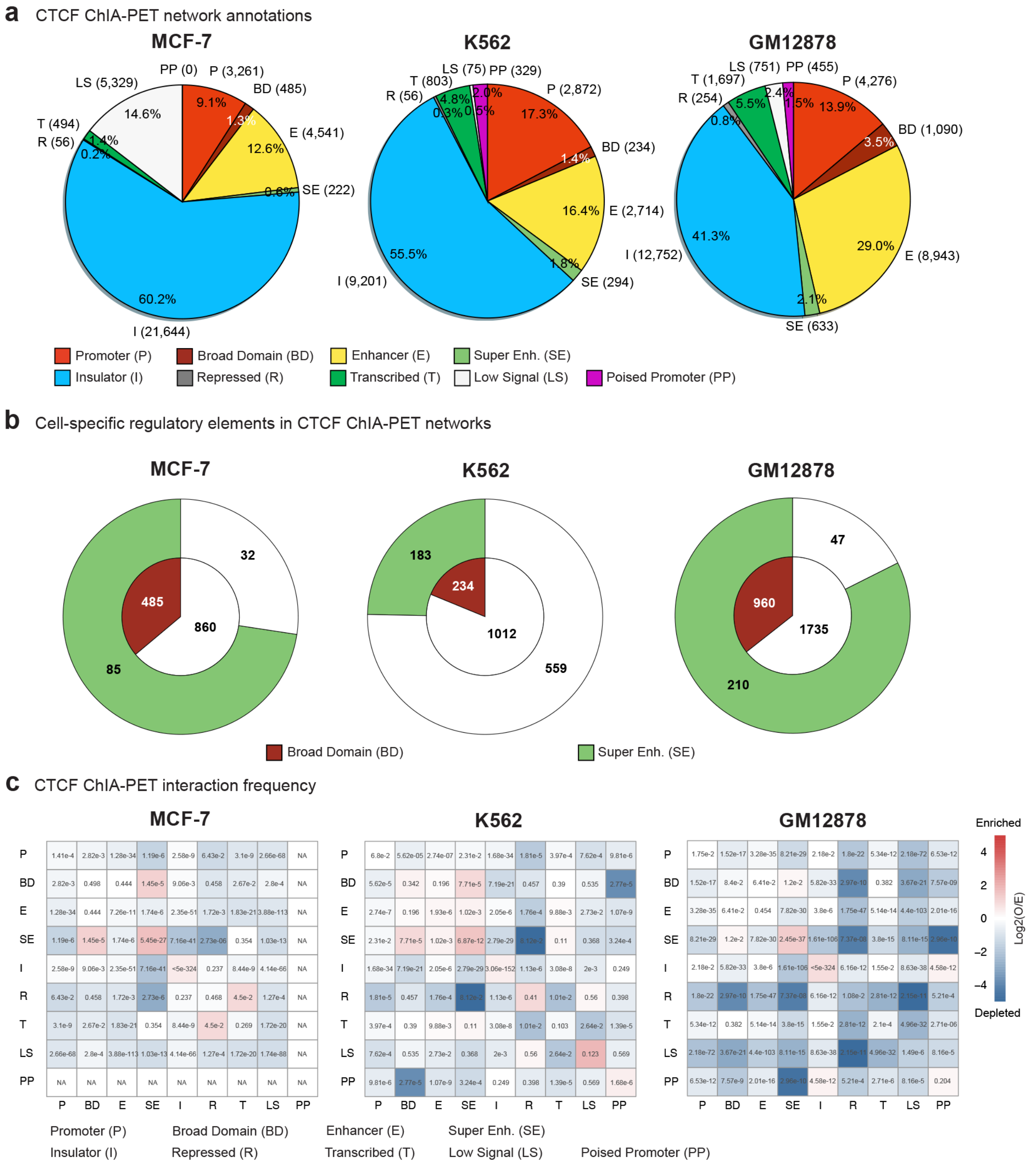

Supplementary Figure 2. Representation of regulatory elements and interaction frequencies in CTCF-mediated ChIA-PET networks. (a) Distribution of different annotations. **(b)** Distribution of broad domains (inner chart) and super enhancers (outer chart). **(c)** Interaction frequency matrices between pairs of annotation classes based on the log2 ratio of observed over expected number of edges between nodes with the respective annotations. Values within each cell represent one tailed binomial test p-values (greater than or less than hypothesis depending on the ratio). Note that CTCF networks do not capture as many regulatory elements as Pol2 networks, therefore Pol2 ChIA-PET data are used in the rest of our analyses.

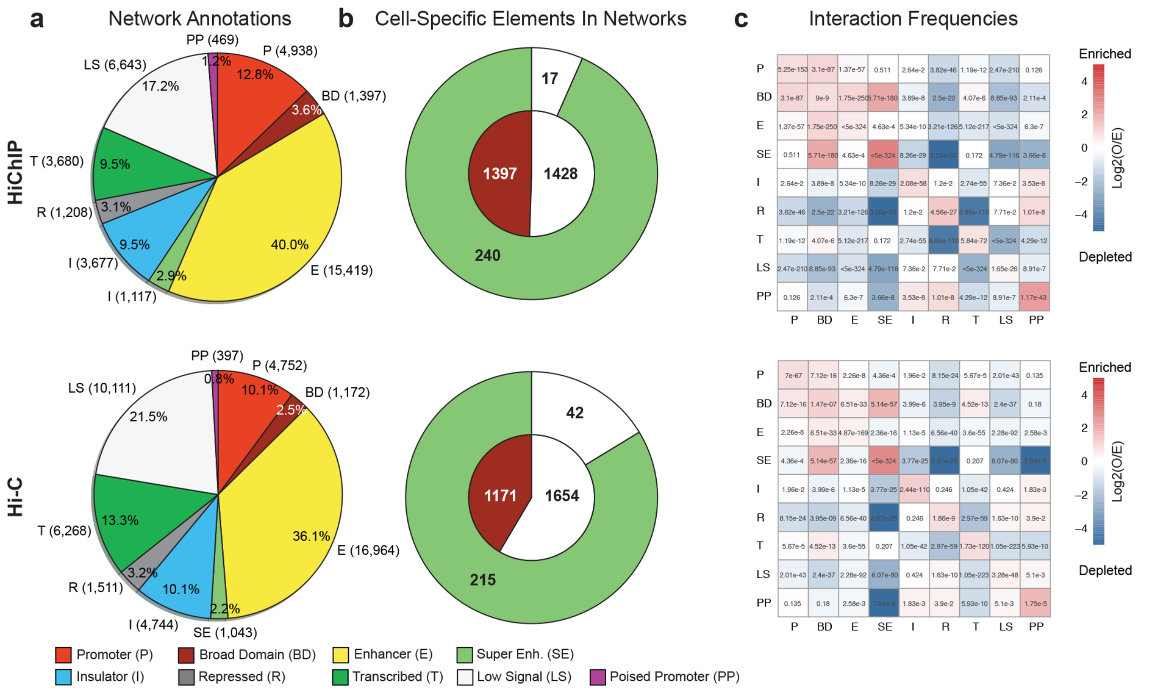

Supplementary Figure 3. Representation of regulatory elements and interaction frequencies in Hi-ChIP and Hi-C networks. (a) Distribution of different annotations in Hi-ChIP (top) and Hi-C (bottom) networks in GM12878. **(b)** Distribution of broad domains (inner chart) and super enhancers (outer chart) in HiChIP (top) and Hi-C (bottom) networks in GM12878. **(c)** Interaction frequency matrices between pairs of annotation classes in Hi-ChIP (top) and Hi-C (bottom) networks in GM12878 based on the log2 ratio of observed over expected number of edges between nodes with the respective annotations. Values within each cell represent one tailed binomial test p-values (greater than or less than hypothesis depending on the ratio).

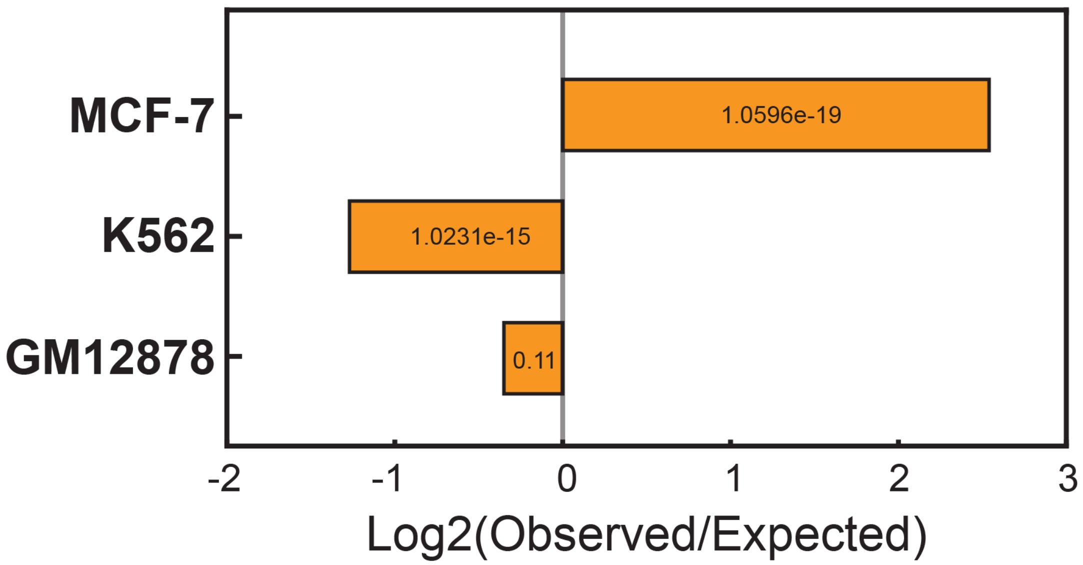

Supplementary Figure 4. Interaction enrichment of merged Super Enhancer Nodes in Pol2 ChIA-PET networks. Interaction frequency of super enhancers after merging super enhancer nodes belonging to the same super enhancer region. Values within each bar represent one tailed binomial test p-values (greater than or less than hypothesis depending on the ratio). Note that the enrichment is lost after the merging except in MCF7.

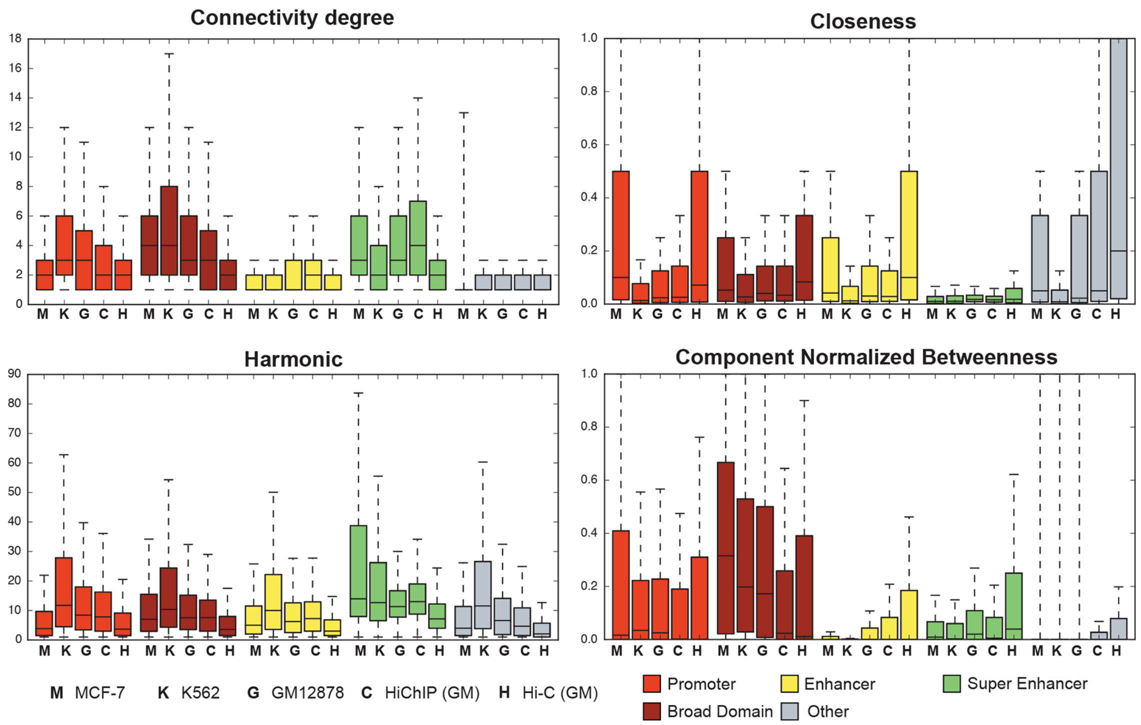

Supplementary Figure 5. Centrality Score Distributions. Distribution of centrality scores (connectivity degree, closeness, harmonic, normalized betweenness centrality) for different annotation classes. M, K, G represents MCF-7, K562, and GM12878 Pol2 ChIA-PET networks respectively. C and H represent HiChIP and Hi-C GM12878 networks respectively.

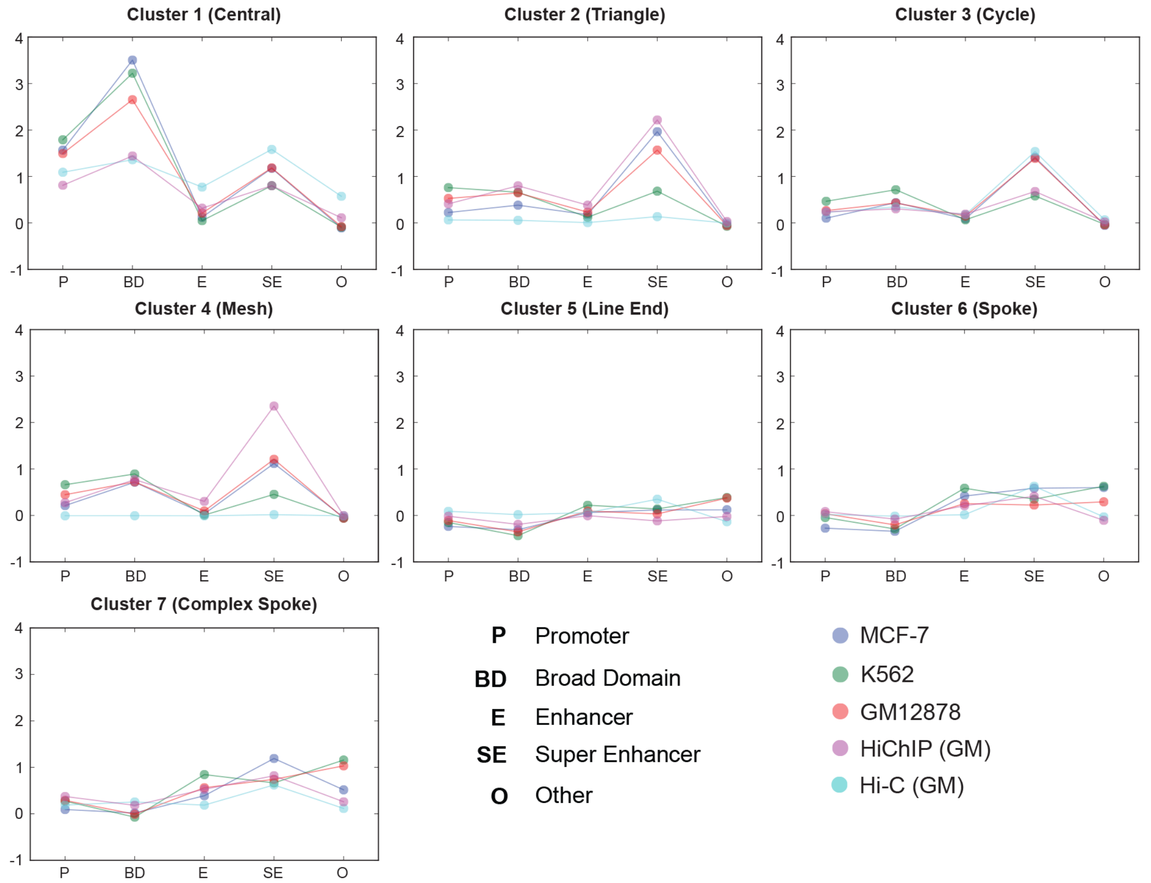

Supplementary Figure 6. Seven orbit cluster scores for different regulatory elements. Trimmed mean cluster scores for ChIA-PET (MCF-7, K562, GM12878), Hi-ChIP (GM12878), and Hi-C (GM12878) networks for promoters (P), broad domains (BD), enhancers (E), super enhancers (SE) and other regulatory elements (O). Note that these measures are very similar across cell types and assays.

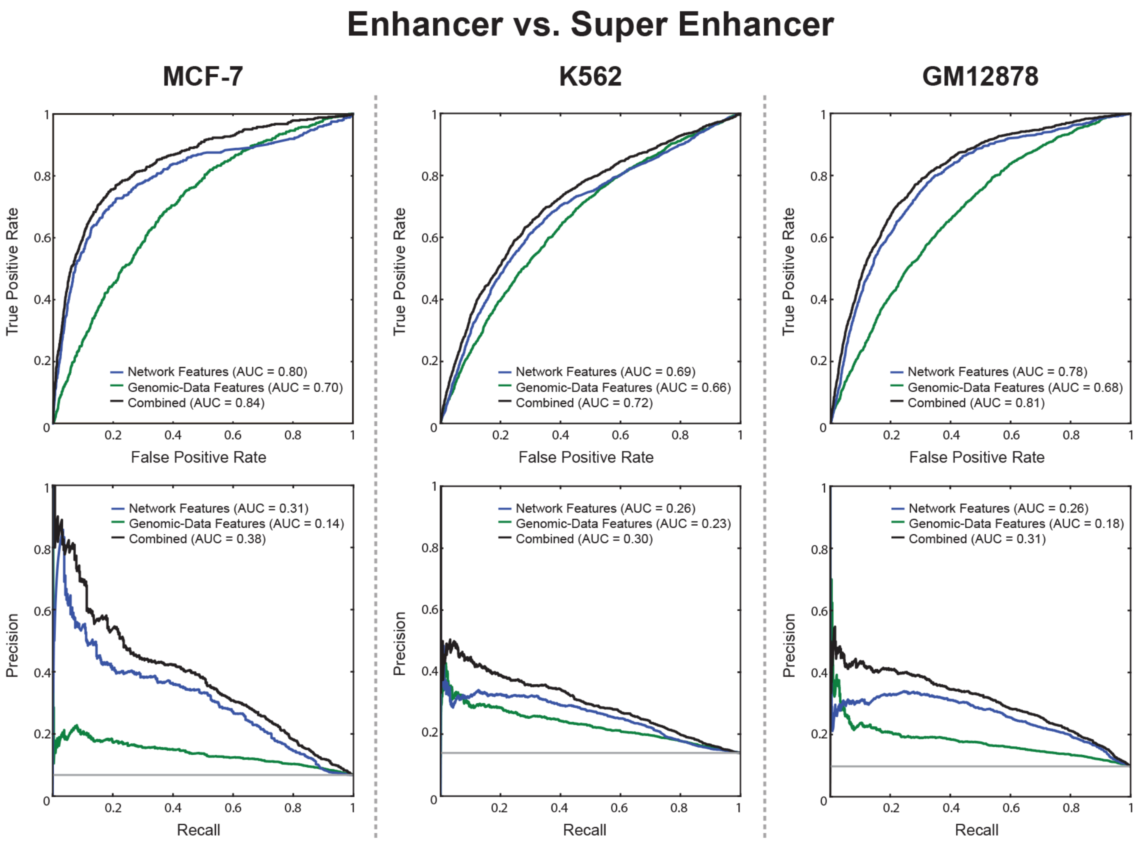

Supplementary Figure 7. SVM models to discriminate enhancers and super enhancers using Pol2 ChIA-PET data. Receiver Operating Characteristic (ROC) curves (top) and precision recall curves (bottom) for SVM models separating enhancers from super enhancers using ChIA-PET networks (baseline performance shown in gray). AUC: area under the curve. Colors represent different data features used in the models.

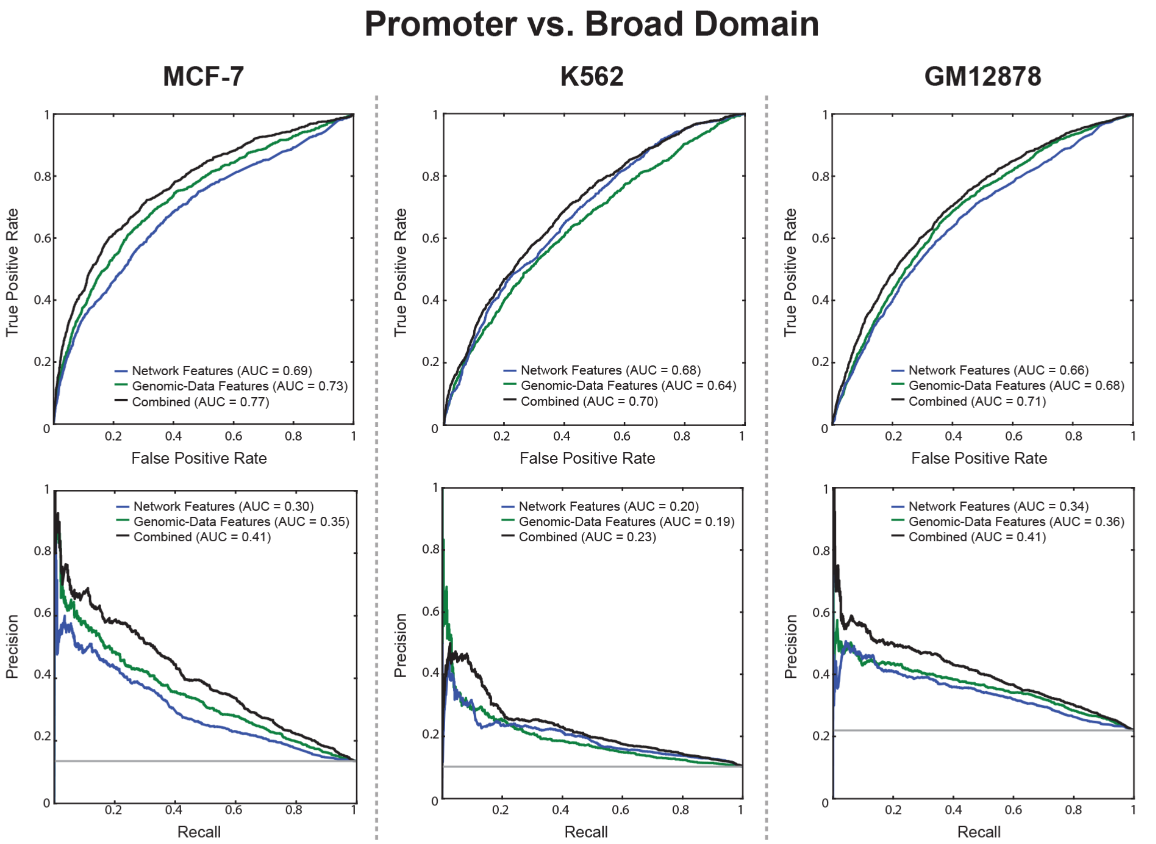

Supplementary Figure 8. SVM models to discriminate promoters and broad domains using Pol2 ChIA-PET data. Receiver Operating Characteristic (ROC) curves (top) and precision recall curves (bottom) for SVM models separating promoters from broad domains for ChIA-PET networks (baseline performance shown in gray). AUC: area under the curve. Colors represent different data features used in the models.

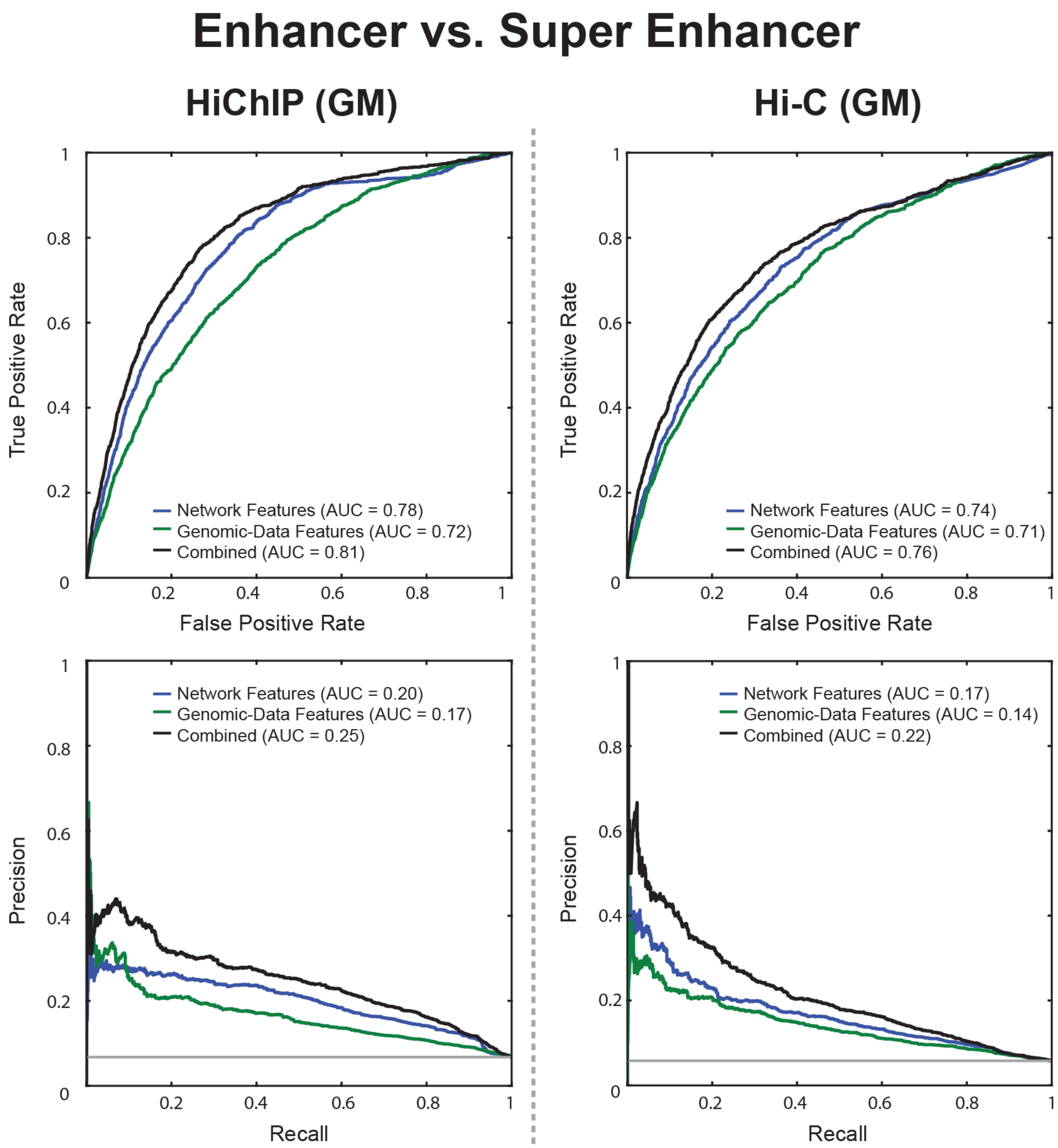

Supplementary Figure 9. SVM models to discriminate enhancers and super enhancers using HiChIP and Hi-C data. Receiver Operating Characteristic (ROC) curves (top) and precision recall curves (bottom) for SVM models separating enhancers from super enhancers for HiChIP (left) and Hi-C (right) GM12878 networks (baseline performance shown in gray). AUC: area under the curve.

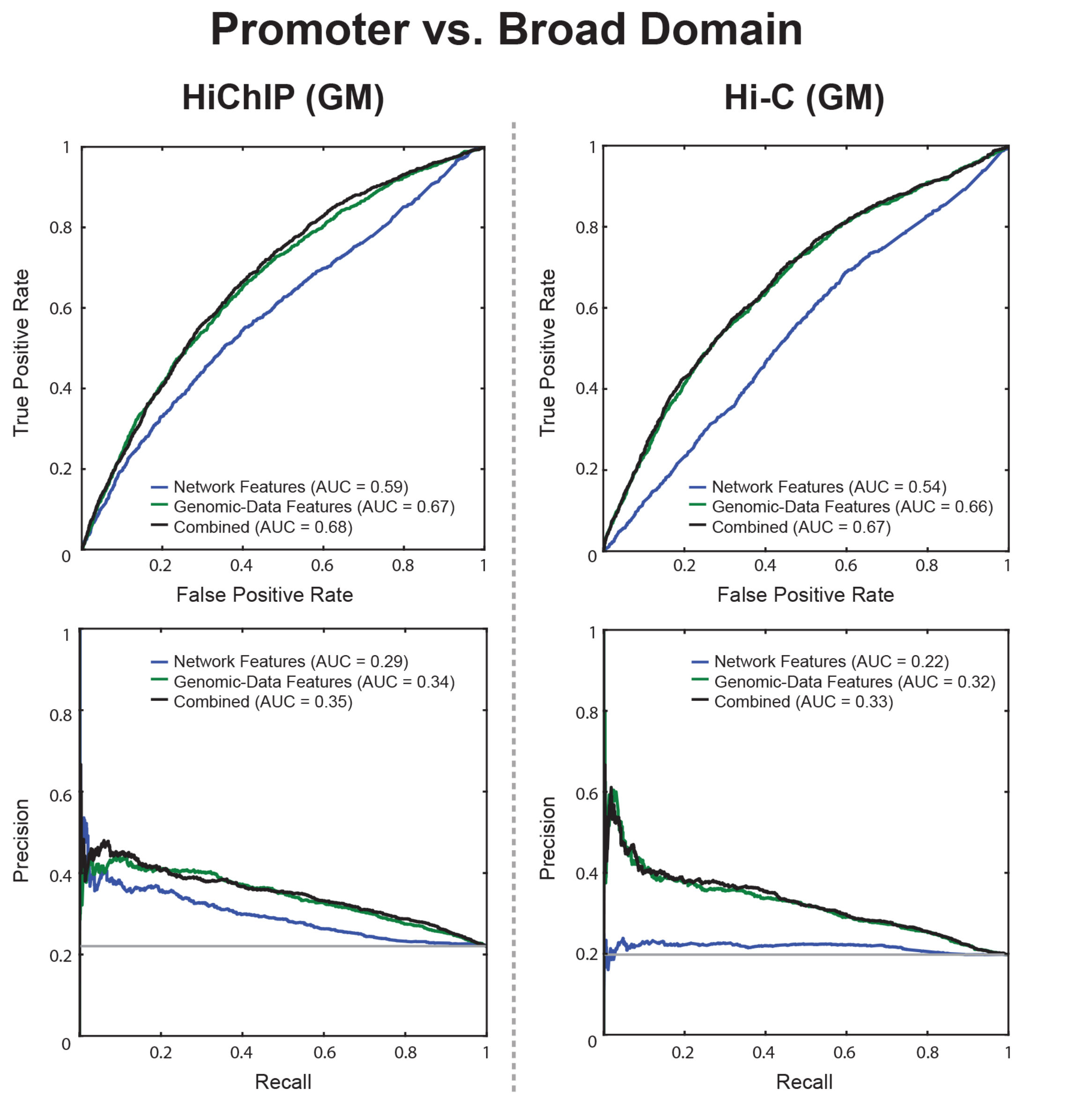

Supplementary Figure 10. SVM models to discriminate promoters and broad domains using HiChIP and Hi-C data. Receiver Operating Characteristic (ROC) curves (top) and precision recall curves (bottom) for SVM models separating promoters from broad domains for HiChIP (left) and Hi-C (right) GM12878 networks (baseline performance shown in gray). AUC: area under the curve.

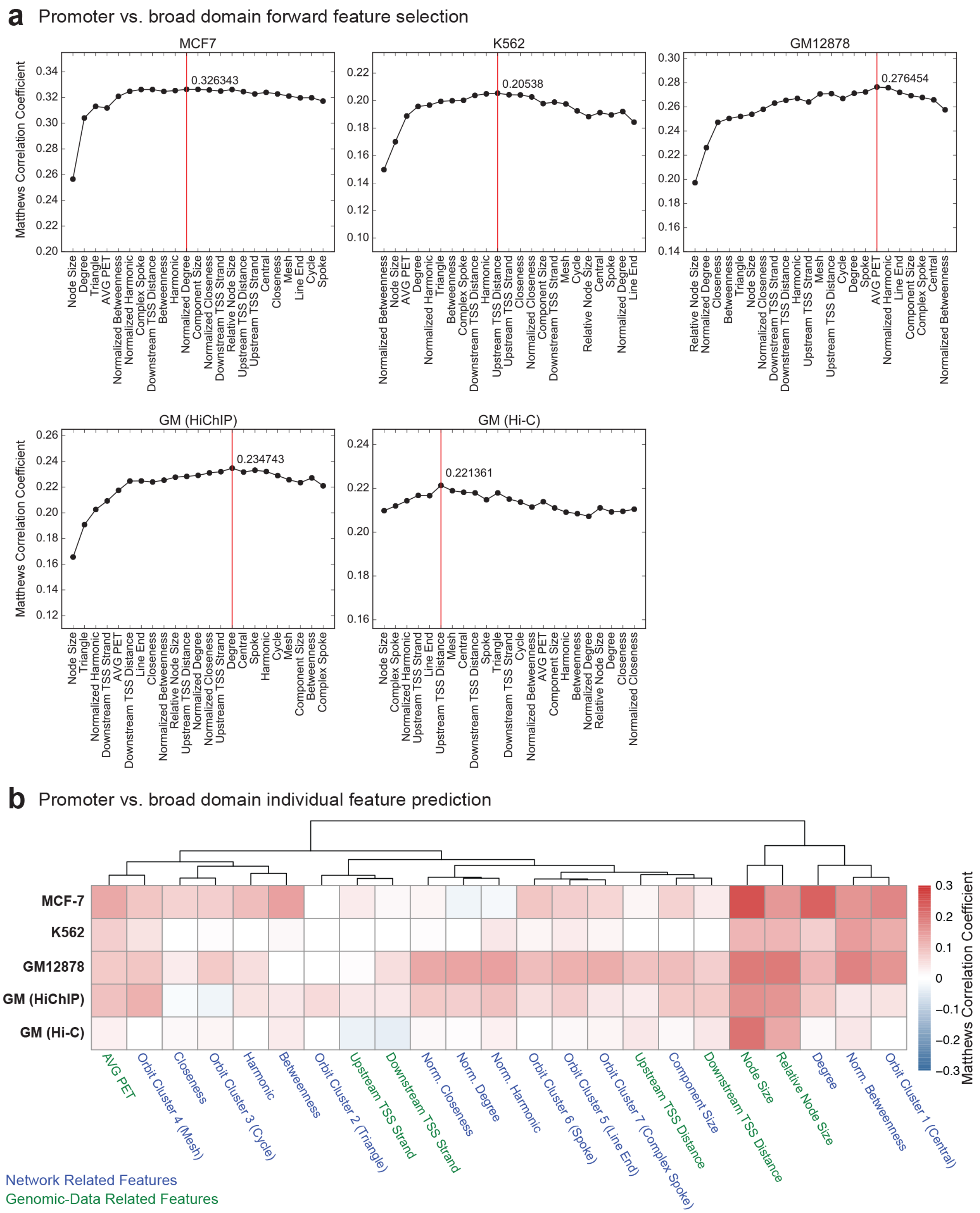

Supplementary Figure 11. Feature ranking for SVM models to discriminate promoters and broad domains. (a) Feature importance ranking for all networks derived from forward selection for broad domain prediction. Forward selection incrementally includes features with the highest Matthews correlation coefficient score in each step. **(b)** Performance of training models with individual features. Features are divided into network related features (blue labels) and genomic-data related features (green labels). Note that the most predictive features for broad domains are node size, and centrality related measures (degree, cluster 1 score).

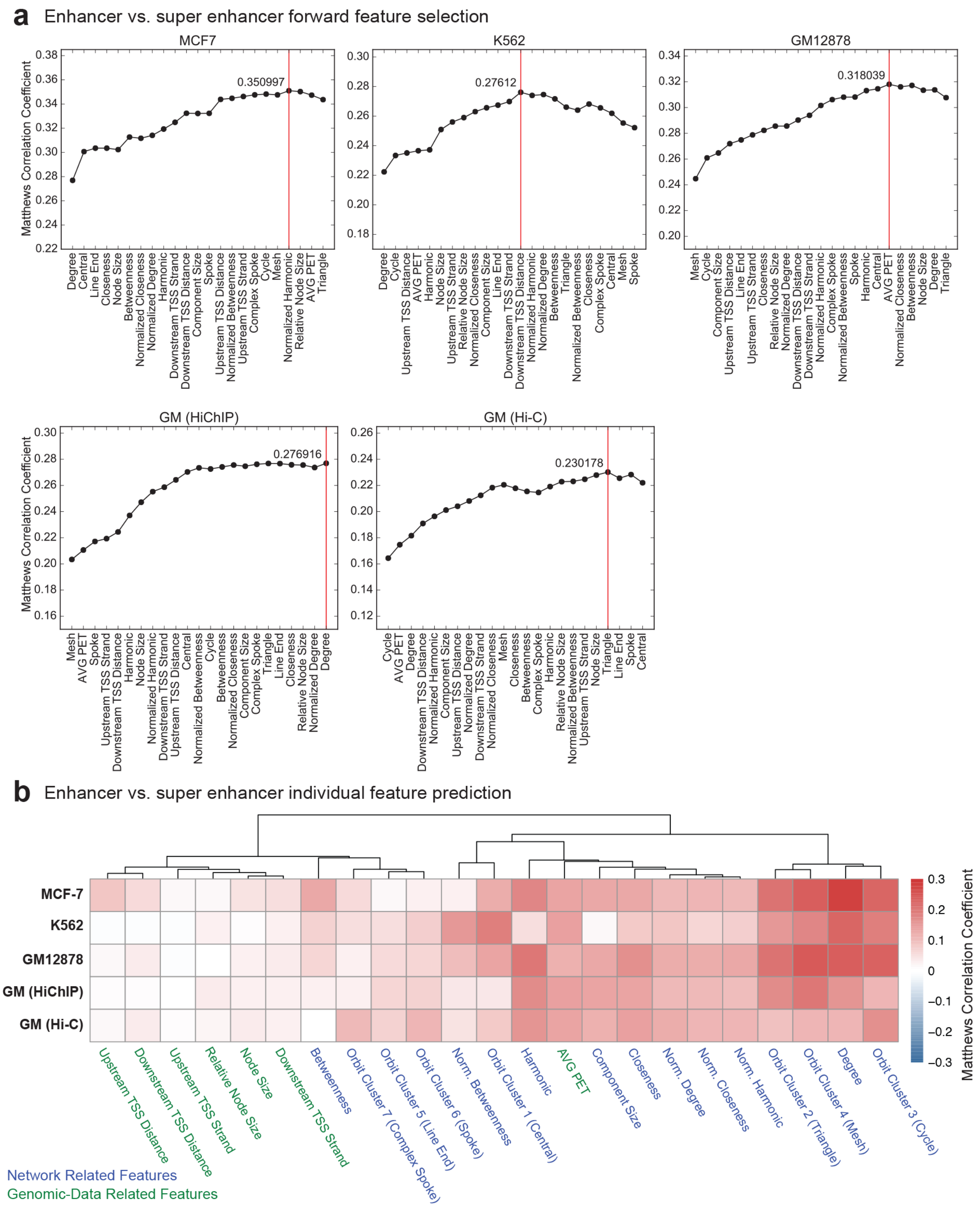

Supplementary Figure 12. Feature ranking for SVM models to discriminate enhancers and super enhancers. (a) Feature importance ranking for all networks derived from forward selection for super enhancer prediction. Forward selection incrementally includes features with the highest Matthews correlation coefficient score in each step. **(b)** Performance of training models with individual features. Features are divided into network related features (blue labels) and genomic-data related features (green labels). Note that the most important features are associated with clique-like patterns associated with super enhancers.

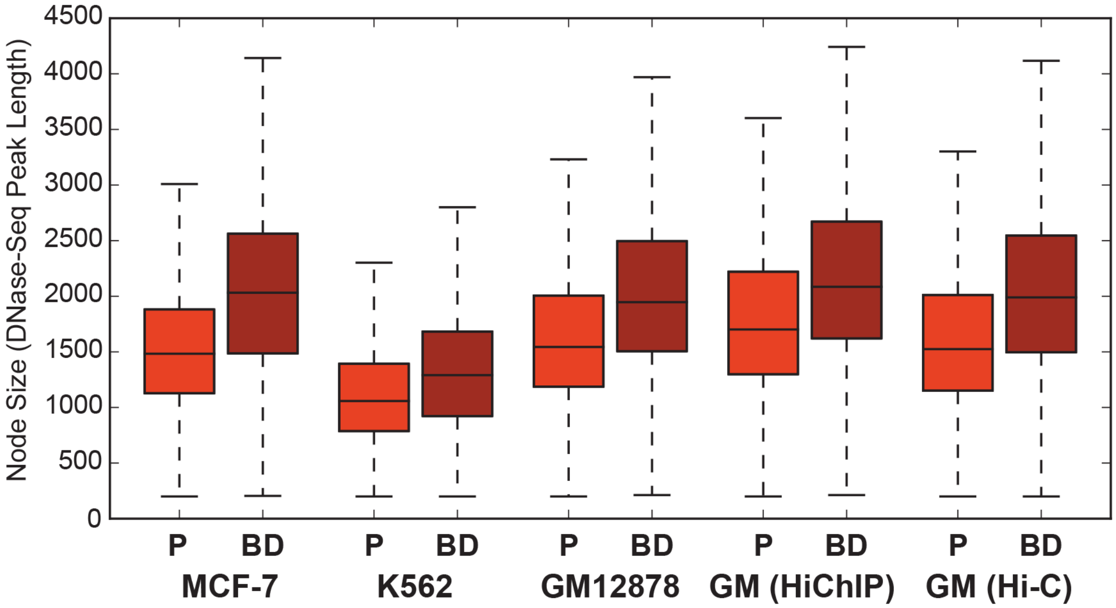

Supplementary Figure 13. Node size of promoters and broad domains. Node size (Dnase-seq peak length) comparison between promoters (P) and broad domains (BD) within MCF-7, K562, and GM12878 ChIA-PET, GM12878 HiChIP, and GM12878 Hi-C networks.
